# Congenital Heart Diseases Impair Female Fertility

**DOI:** 10.3389/fped.2021.687276

**Published:** 2021-07-14

**Authors:** Shao-Ju Chien, Ying-Jui Lin, Mao-Hung Lo, Chien-Fu Huang, Yao-Hsu Yang

**Affiliations:** ^1^Department of Pediatrics, Kaohsiung Chang Gung Memorial Hospital and College of Medicine, Chang Gung University, Kaohsiung, Taiwan; ^2^Department of Early Childhood Care and Education, Cheng Shiu University, Kaohsiung, Taiwan; ^3^Health Information and Epidemiology Laboratory of Chang Gung, Memorial Hospital Chiayi, Chiayi, Taiwan; ^4^Department of Traditional Chinese Medicine, Chang Gung Memorial, Hospital Chiayi, Chiayi, Taiwan; ^5^School of Traditional Chinese Medicine, College of Medicine, Chang Gung University, Taoyuan, Taiwan

**Keywords:** fertility, abortion, congenital heart disease, national health insurance research database, live births

## Abstract

**Background:** The objective of this research was to evaluate the fertility of Taiwanese women with diagnoses of congenital heart diseases (CHDs). The study also investigated how different forms of CHDs may have variously influenced fertility.

**Methods:** We directed this nationwide, population-based and retrospective matched-cohort research by using data from the Taiwan National Health Insurance Research Database. The CHD group (*n* = 6602) included women with congenital structural heart diseases, aged 16–45 years in 2000. The non-CHD group (*n* = 6602) was matched according to urbanization and income. The outcomes, involving live birth, abortion, and fertility rates, were followed until the end of 2013. Poisson regression was used to evaluate the incidence rate ratios (IRRs).

**Results:** The CHDs had an inferior rate of live births (IRR 0.74 [95% CI 0.71–0.78]) than the non-CHD group. There was also a lower fertility rate in the CHD group (IRR 0.81 [95% CI 0.78–0.84]) than the non-CHD group. Abortion rates between the two groups were similar.

**Conclusion:** Congenital structural heart disease compromises female fertility, even among patients with simple forms of CHDs. It is suggested that pregnant patients with CHDs are early appeared to and advised personally with multidisciplinary care to improve their outcomes.

## Introduction

As a result of advances in cardiac surgery and pediatric medical care, more children with congenital heart diseases (CHDs) have reached grown-up in the past decades, than in any history. Only 20%−30% of children with CHDs survived into parenthood 50 years ago, but, today, over 90% survive long-term. In turn, the adult CHDs population is steadily growing, even for patients with the most complex lesions (1, 2). According to Wu's study, based on the Taiwan National Health Insurance Research Database, the event-free survival rate (95% confidence interval [95% CI]) of all children with CHDs in Taiwan was 95.1% (94.5–95.4%). Even among patients with severe CHDs, the overall survival rate was 76.4%, with survival rates of 87.8% and 66.7% for tetralogy of Fallot and transposition of the great arteries patients, respectively (3). An increasing number of women with CHDs thrive well into their childbearing years. Expecting increased quality of life, affected women often express a desire to start a family, containing giving birth to children, despite possible cardiac complications caused by the hemodynamic alterations associated with pregnancy (4). Although the majority of pregnancies do not lead to major complications in women with CHDs, the amount of risk for both mother and newborn increases concurrently with the complexity of the underlying heart anomaly (2, 5, 6). Pregnancy will hazard their life of women with CHDs with an increased risk (18%) of fetal and neonatal complications in comparison with healthy women (7%) (7).

The 2011 guidelines of the European Society of Cardiology on the management of cardiovascular diseases during pregnancy, sketch some risk factors of neonatal miscarriage and perinatal mortality that include maternal structural heart diseases (8–11). However, it is unknown whether maternal CHDs is associated with more miscarriages or stillbirths. Besides, women with pulmonary hypertension have a high risk during pregnancy and associated with high morbidity and mortality in all defined clinical groups of pulmonary hypertension (12). The purpose of this nationwide retrospective cohort study was to investigate the fertility rate of women with CHDs, focusing on their live births, abortions, and fertility. We also investigated how different forms of CHDs and the presence of pulmonary hypertension may have variously influenced the observed fertility rate. To our knowledge, the current study is the first to provide an impression of live birth, abortion, and fertility rates, based on data from a large, national database of women with CHDs, and to include a comparison of the different forms of CHDs.

## Methods

### Source of Datasets

The research data were retrieved from the National Health Insurance Research Database (NHIRD). Taiwan government launched a single-payer National Health Insurance (NHI) program on March 1, 1995 and the coverage rate is around 99% of the population by the end of 2014. The NHIRD contains all de-identified registration files and original medical claims data from the NHI program. The completeness and accuracy of NHIRD have been reported and acceptable, and it has been used in previous research of CHDs (3). This study was approved by the Institutional Review Board of Chang Gung Medical Foundation (IRB No. 1906140058).

### Matched Cohort Design

We searched for female patients with CHDs in the NHIRD across the 1997–2013 range according to the International Classification of Diseases 9th Revision, Clinical Modification [ICD-9-CM] codes of 745.xx, 746.xx, 747.xx to 747.49. Patients with CHDs also had catastrophic illness/injury certificates (CICs). Taiwan National Health Insurance Administration issues a CIC to patients with CHDs except some insignificant or spontaneously resolved cardiac defects. Cardiologists or pediatric cardiologists approve the CHD diagnosis according to patients' clinical manifestations and echocardiographic reports before issuing a CIC. The research excluded patients younger than 16 years or older than 45 years in 2000 to ensure that the study subjects were entirely within reproductive age during the research period. The date when the CIC was issued was considered as the date of a diagnosis of CHD.

**Figure 1:**
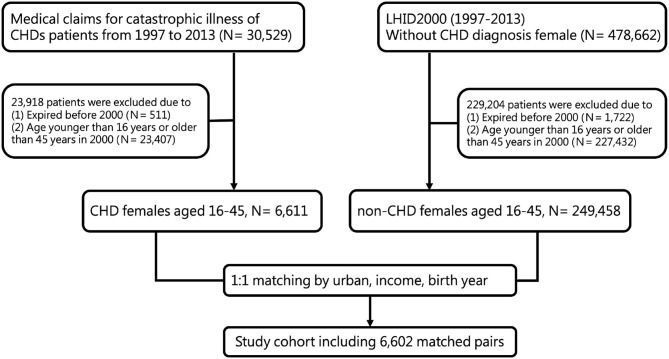
The flow chart of case selection and exclusion of women with a history of CHDs.

Matched subjects, without CHDs, were found by searching the Longitudinal Health Insurance Database of 2000 (LHID-2013). These subjects were matched one-to-one with CHD subjects for age, gender, income, and urbanization rate. The LHID is a subset of the NHIRD and contains 1,000,000 randomly sampled beneficiaries from 2000. It constitutes about 5% of Taiwan's population and its gender distribution does not differ significantly from that of the NHIRD. Incomes were classified into four groups: 0, 1–15840, 15840–25000, and >25000 New Taiwan dollars per month. Urbanization levels were classified as very high, high, moderate, and low. The accessibility and availability of health care will be affected by these factors. Female infertility (ICD-9-CM code 628) listed in the leading five diagnoses in any outpatient or inpatient medical records was recognized as a confounding factor. The flow chart of case selection and exclusion is shown in Figure 1.

### Outcomes and Risk Factors

The study outcomes involved live birth, abortion, and fertility rates. Live birth was defined as a normal vaginal delivery in the inpatient diagnosis with or without complications (NHI codes: 97001A, 97001K, 97002A, 97003B, 97004C, 97005D, 97931K, 97932A, 97933B, 97934C, 81017C, 81024C, and 81034C). Besides a cesarean delivery (NHI codes: 97006K, 97007A, 97008B, 97009C, 97014C, 98001K, 98002A, 98003B, 98004C, 81004C, 81005B, 81005C, 81028C, and 81029C), or a multifetal pregnancy (NHI codes: 81018C, 81019C, 81025C, and 81026C) were also included (13). Abortion was defined as ICD-9-CM codes of 632, 633, 634, and 637 in the first five diagnoses of inpatient and ambulatory claims (14). The diagnosis of abortion with an interval in excess of 1 month and 10 months for an ectopic pregnancy were regarded as separate events. Total numbers of live births and abortions was defined as fertility. Outcomes were followed until the end of the study period or death.

Various cardiac structural defects were also investigated as potential factors affecting fertility rates. The recommended parameters from the American College of Cardiology were used to classify levels of disease severity. According to the college's modified forms, simple CHD patients are those who can typically be cared for by the general medical community, while patients with severe CHDs are those who should be seen regularly at centers with physicians experienced in administering care for CHD patients (15). Because the 2011 guidelines of the European Society of Cardiology define maternal left heart obstruction and single ventricle physiology as high or very high risk factors for neonatal complications (10), the left-sided heart lesions and single ventricle groups were all classified into severe CHD group for data analysis. To avoid errors caused by incorrect tentative diagnosis and miscoding, we searched for CHD diagnoses in the top five diagnoses of inpatient claims among those patients with CICs of CHDs. All CHDs were classified into the two forms according to their cardiac structural defects. According to Wu's Taiwanese national population studies in 2010 and 2015, the codes encompassing “simple” CHDs, included secundum atrial septal defects (7455), ventricular septal defects (7454), patent ductus arteriosus (7470), pulmonary stenosis/anomalies (74602), and partial anomalous pulmonary venous connections (74742). The diagnostic codes comprising “severe” CHD were common truncus (7450, 74500), transposition of great vessels/congenitally corrected transposition of great arteries (7451, 74510, 74512), tetralogy of Fallot (7452), common ventricle (7453), endocardial cushion defects (7456, 74560, 74569), anomalies of pulmonary valve/artery (7460, 7473, 74600, 74601, 74609), tricuspid atresia and stenosis (7461), Ebstein's anomaly (7462), congenital stenosis or insufficiency of mitral or aortic valve (7463, 7464, 7465, 7466), subaortic stenosis (74681), coarctation of the aorta or anomalies of aorta (7471, 74710, 74711, 7472, 74720, 74721, 74722, 74729), double outlet right ventricle (74511), common atrium (74561), cor triatriatum (74682), infundibular pulmonic stenosis (74683), coronary artery anomaly (74685), congenital heart block (74686), total anomalous pulmonary venous return (74741) and hypoplastic left heart syndrome (7467) (3, 16). Pulmonary hypertension was defined as ICD-9-CM codes of 4160, 4168 and 4169 (17).

### Statistical Analyses

The distribution of demographic data and the proportions of different CHDs were compared between patients with and without CHDs. We calculate the incidence rate per 10,000 person-years as IR. Poisson regression was used to assess incidence rate ratios (IRRs) and the corresponding 95% confidence interval (CI) of live births, abortions, and fertility rates between the patients with and without CHDs. To investigate the influence of different structural defects, severity levels and pulmonary hypertension on fertility in female patients with CHDs, we compared IRRs within the CHD groups, according to their structural complexity and association. All analyses were conducted by means of SAS statistical software (version 9.4; SAS Institute, Cary, NC, USA).

## Results

### Characteristics of the Study Subjects

The characteristics and comparisons of the 6,602 study subjects and 6,602 age-, income-, and urbanization-matched non-CHD subjects from the LHID are shown in Table 1. We also regarded their underlying infertility probably as confounding factor, their diagnoses of female infertility in past medical records were also compared without significant differences between the CHD and non-CHD groups. Fertility frequencies of 0, 1, 2, and ≥3 in the CHD and non-CHD groups during the research period were 66, 15, 12, and 8% vs. 59, 16, 14, and 11%, respectively.

**Table 1:** Characteristics of the study subjects.

**Variables**	**CHD**		**Non-CHD**		***p*-value**
	**(*****N*** **= 6,602)**		**(*****N*** **= 6,602)**		
	***n***	**%**	***N***	**%**	
Urbanization level					1.000
Very high	2,151	33%	2,151	33%	
High	3,063	46%	3,063	46%	
Moderate	944	14%	944	14%	
Low	444	7%	444	7%	
Income (NTD)					1.000
0	2,953	45%	2,953	45%	
1–15840	1,122	17%	1,122	17%	
15841–25000	1,921	29%	1,921	29%	
>25000	606	9%	606	9%	
Comorbidities					0.100
Female infertility					
Yes	330	5%	290	4%	
No	6,272	95%	6,312	96%	
Fertility					<0.001
0	4,332	66%	3,902	59%	
1	981	15%	1,073	16%	
2	765	12%	892	14%	
≧3	524	8%	735	11%	

### Live Births, Abortions, and Fertility Rates of CHD and Non-CHD Patients

The study population was composed of 6,602 patients who were diagnosed with CHDs and were 16–45 years of age. The comparison of live births, abortions and fertility rate between CHD and non- CHD patients are demonstrated in Table 2. Of these women, 2,270 had a total of 2,835 live births. There were fewer live births in CHD patients (IRR 0.74, 95% CI 0.71–0.78, *p* < 0.001). The number of abortions was comparable between the CHD and non-CHD groups. As a result, fertility was inferior in the CHD group (IRR 0.81, 95% CI 0.78–0.84, *p* < 0.001).

**Table 2:** Live births, abortions, and fertility of CHD and non-CHD.

	**CHD (*****N*** **= 6,602)**					**Non-CHD (*****N*** **= 6,602)**					**IRR**	**95% CI**		***p*-value**
	**No. of events**	**PY**	**IR**	**95% CI**		**No. of events**	**PY**	**IR**	**95% CI**					
Live births	2,835	90523.1	313.2	301.9	324.9	3,898	92130.272	423.1	410.0	436.6	0.7402	0.7053	0.7769	< 0.001^*^
Abortions	1,566	90523.1	173.0	164.6	181.8	1,626	92130.272	176.5	168.1	185.3	0.9802	0.9145	1.0506	0.572
Fertility	4,401	90523.1	486.2	472.0	500.8	5,524	92130.272	599.6	584.0	615.6	0.8109	0.7794	0.8436	<0.001^*^

*IR, incidence rate per 10,000 person-years; IRR, incidence rate ratio, comparing CHD cohort with the control cohort; PY, person years*.

* *Statistically significant*.

### Distribution of Forms of Congenital Heart Diseases

The underlying congenital heart lesions, the number of patients affected by each, the associated ICD-9-CM codes, and the groupings are summarized in Table 3. For more accurate analysis of different CHDs on fertility, we only retrieved the ICD-9 codes of definite CHD diagnosis as mentioned in the methods and excluded some uncertain diagnosis with the beginning words “other” or “unspecified”. Some patient might have more than one CHD diagnosis, we grouped patient into simple or severe CHDs according to the most complicated CHD diagnosis she had.

**Table 3:** The distribution of congenital heart diseases.

**CHD type**	**ICD-9-CM**	**Group**	**Numbers**
ASD-II^a^	745, 7455	Simple	2,657
Ventricular septal defect	7454	Simple	1,415
Patent ductus arteriosus	7470	Simple	516
Congenital stenosis of pulmonary valve	74602	Simple	57
PAPVR^b^	74742	Simple	10
Common truncus	7450, 74500	Severe	61
Transposition of great vessels	7451, 74510, 74512	Severe	12
Tetralogy of Fallot	7452	Severe	173
Common ventricle	7453	Severe	14
Endocardial cushion defects	7456, 74560, 74569	Severe	36
Anomalies of pulmonary valve/artery	7460, 7473, 74600, 74601, 74609	Severe	97
Tricuspid atresia and stenosis	7461	Severe	6
Ebstein's anomaly	7462	Severe	51
Aortic valve/subaortic stenosis	7463, 74681	Severe	68
Congenital insufficiency of aortic valve	7464	Severe	85
Congenital mitral stenosis/insufficiency	7465, 7466	Severe	48
Coarctation of aorta/anomalies of aorta	7471, 74710, 74711, 7472, 74720, 74721, 74722, 74729	Severe	87
Double outlet right ventricle	74511	Severe	6
Common atrium	74561	Severe	35
Cor triatriatum	74682	Severe	7
Infundibular pulmonic stenosis	74683	Severe	76
Coronary artery anomaly	74685	Severe	75
Congenital heart block	74686	Severe	12
TAPVR^c^	74741	Severe	5

a *Ostium secundum type atrial septal defect*.

b *Partial anomalous pulmonary venous connection*.

c *Total anomalous pulmonary venous connection*.

### Influence of Varying Severity Levels of CHDs on Live Birth, Abortion, and Fertility Rates

The influence of different CHD severity on live births, abortions, and fertility are illustrated in Table 4. There were significantly fewer live births and a significantly lower fertility rate for all CHDs, as compared to non-CHD patients, but the abortion rate did not differ between the groups. A trend was observed in which the live birth rate decreased with increasing CHD severity and complexity, for these two CHD classifications.

**Table 4:** The influence of different cardiac structural defects on live births, abortions, and fertility.

**Group**	**Live births**				**Abortions**				**Fertility**			
	**IRR**	**95% CI**		***p*-value**	**IRR**	**95% CI**		***p*-value**	**IRR**	**95% CI**		***p*-value**
Simple CHDs	0.79	0.74	0.84	<0.0001^*^	1.01	0.93	1.10	0.859	0.85	0.81	0.90	<0.0001^*^
Severe CHDs	0.60	0.52	0.69	<0.0001^*^	1.04	0.86	1.27	0.688	0.72	0.64	0.80	<0.0001^*^

*IR, incidence rate per 10,000 person-years; IRR, incidence rate ratio, comparing CHD cohort with the control cohort; PY, person years*.

* *Statistically significant*.

### Influence of Pulmonary Hypertension of CHDs on Live Birth, Abortion, and Fertility Rates

We further evaluated the influence of pulmonary hypertension of CHDs on live birth, abortion, and fertility rates in Table 5. During the study period, CHDs with pulmonary hypertension had a significantly lower rate of live births (IRR 0.83, 95% CI 0.73–0.93, *p* = 0.002) than CHDs without pulmonary hypertension, and this trend was even lower among the severe CHDs group combined with pulmonary hypertension (IRR 0.63, 95% CI 0.40–0.99, *p* = 0.046). The abortion rate was similar between the CHDs no matter if they were simple or severe groups, nor they had pulmonary hypertension or not. Similar to live births, fertility was lower in the simple CHDs with pulmonary hypertension (IRR 0.87, 95% CI 0.78–0.97, *p* = 0.014). However, severe CHDs with pulmonary hypertension didn't demonstrate significantly different fertility as compared to those without pulmonary hypertension (IRR 0.74, 95% CI 0.53–1.03, *p* = 0.074).

**Table 5:** The influence of pulmonary hypertension on live births, abortions, and fertility among different CHDs.

	**CHDs with Pulmonary hypertension (*****N*** **= 868)**					**CHDs without Pulmonary hypertension (*****N*** **= 5,734)**					**IRR**	**95% CI**		***p*-value**
	**No. of events**	**PY**	**IR**	**95% CI**		**No. of events**	**PY**	**IR**	**95% CI**					
Live births	301	11370.1	264.7	236.5	296.4	2,534	79153.0	320.1	307.9	332.9	0.83	0.73	0.93	0.002^*^
Abortions	192	11370.1	168.9	146.6	194.5	1,374	79153.0	173.6	164.6	183.0	0.97	0.84	1.13	0.720
Fertility	493	11370.1	433.6	397.0	473.6	3,908	79153.0	493.7	478.5	509.5	0.88	0.80	0.96	0.007^*^
Group1. Simple CHDs														
	**Pulmonary hypertension (*****N*** **= 640)**					**No Pulmonary hypertension**					**IRR**	**95% CI**		***p*****-value**
	**No. of events**	**PY**	**IR**	**95% CI**		**No. of events**	**PY**	**IR**	**95% CI**					
Live births	233	8451.9	275.7	242.5	313.4	1,714	51612.3	332.1	316.7	348.2	0.83	0.72	0.95	0.008^*^
Abortions	144	8451.9	170.4	144.7	200.6	922	51612.3	178.6	167.5	190.6	0.95	0.80	1.14	0.597
Fertility	377	8451.9	446.1	403.2	493.4	2,636	51612.3	510.7	491.6	530.6	0.87	0.78	0.97	0.014^*^
Group2. Severe CHDs														
	**Pulmonary hypertension (*****N*** **= 87)**					**No Pulmonary hypertension**					**IRR**	**95% CI**		***p*****-value**
	**No. of events**	**PY**	**IR**	**95% CI**		**No. of events**	**PY**	**IR**	**95% CI**					
Live births	20	1122.7	178.1	114.9	276.1	262	9256.7	283.0	250.8	319.5	0.63	0.40	0.99	0.046^*^
Abortions	18	1122.7	160.3	101.0	254.5	162	9256.7	175.0	150.0	204.1	0.92	0.56	1.49	0.724
Fertility	38	1122.7	338.5	246.3	465.2	424	9256.7	458.0	416.5	503.8	0.74	0.53	1.03	0.074

*IR, incidence rate per 10,000 person-years; IRR, incidence rate ratio, comparing CHD cohort with the control cohort; PY, person years*.

* *Statistically significant*.

## Discussion

It is estimated that 12–15% of all pregnancies result in miscarriages, in healthy women (2, 18). For women with CHDs, miscarriages have been observed in 6–15% of all pregnancies (2, 7–9, 19). Stillbirths occur much less commonly than miscarriages. In 2009, the WHO estimated that 0.2–4.7% (average: 2%) of all pregnancies, worldwide, result in stillbirths (20). According to a single-center study in Greece, the offspring mortality rate was 3.7% higher in the CHD mothers than in the general population (21). Other studies have reported higher rates still—up to 16.8%—of miscarriages and stillbirths in women with CHDs (2, 7, 8, 18). Nevertheless, it should be noted that precise numbers are obscure because a high percentage of clinically imperceptible miscarriages occurred during early pregnancy in both patient groups.

In this retrospective research of Taiwanese women of reproductive age with CHDs, we discovered those women had lower live birth and fertility rates. Both rates were even minor among the greater CHD severity levels. The observed rates are comparable to a previous study, conducted in Germany, Hungary, and Japan, which reported an overall miscarriage/stillbirth rate of 24.8%, with no significant difference between pregnancies but with different forms of maternal CHDs (22). However, we evaluated the occurrence of giving birth, not the actual numbers of offspring. It is entirely possible that along with the growing number of mothers with CHDs desiring to become pregnant, the numbers of severe and very severe CHD mothers might also rise. In turn, this may lead to a growing number of neonatal complications, including miscarriages and stillbirths.

There is no doubt that pulmonary hypertension is associated with high morbidity and mortality during pregnancy in the literature review (23). However, some large series didn't identify pulmonary hypertension as a predictor of adverse outcome of maternal complications during pregnancy (9, 19). This is probably due to low prevalence since women with pulmonary hypertension are generally advised against pregnancy, not to mention severe CHDs with pulmonary hypertension. Severe CHD groups had more complicated hemodynamic disturbances and those could further contribute their worse prognosis rather than pulmonary hypertension only. Those were probably the reasons of no significant difference of fertility between our severe CHD group with or without pulmonary hypertension.

The observed abortion rate was comparable between the CHD and non-CHD groups. In Marc-André Koerten's study, only 8% of the observed pregnancies were terminated (22) and around 5–8% were found to be terminated in other literature (2, 8, 24). The overall rate of pregnancy termination in CHD women was lower than that for the general public (30 and 22%, respectively). The difference may be attributable to an intensely strong desire to have a child and/or to their fear about future ability to become pregnant or to give birth among women with CHDs.

We observed that the incidence rate of live births and fertility decreased in CHD patients. These findings might be attributable to not only potential effect of CHD itself or associated underlying genetic problems, other extra-cardiac anomalies and medical therapy like anticoagulants on subsequent fertility but also an inability to tolerate childbearing either due to physician recommendation or impact of CHD on patient's reproductive choice. Considering the psychological issue, patients might be afraid of being pregnant because worry about potential adverse impact brought by CHD and its possible complications such as pulmonary hypertension.

## Limitations

Due to the retrospective database design of the study, it had some limitations. First, the NHIRD does not contain detailed clinical data and, therefore, we could not assess cardiovascular statuses, structure details, and whether patients received any operation/intervention for their cardiac defect. Besides, we didn't analyze any associations of extra-cardiac anomalies and genetic anomalies. It is unknown whether the higher number of stillbirths or abortions is attributable to the occurrence of medical indications for maternal complications or neonatal complications. For the exactitude of the diagnosis entered in the database of the national insurance program and to avoid an overestimation from insignificant or spontaneously resolved cardiac defects, patients with CHDs were enrolled only when they had catastrophic illness certificates. Second, information on marital statuses and educations levels are not obtainable from the NHIRD. Medically assisted procreation was either not taken into consideration for simplification of our focus on the congenital heart disease. Nonetheless, marriage is not an essential condition for fertility and we didn't know the willing of having a child in the study. We couldn't either verify if the infertility was probably related to the partner, rather than the women. We matched CHD patients with non-CHD patients, based on urbanization and income, which are highly associated with health care and education levels. Third, some artificial abortion is not covered by the NHI and performed in local clinics, so the data of self-financed abortion is not retrieved by NHIRD. However, the NHI waives copayment for patients with CHDs and offers affordable care for these patients with CHDs from birth, those CHDs and even complex CHDs should prefer to receive artificial abortion in the official hospital for their safety. These limitations aside, this study is meaningful for a number of reasons: It employed a nationwide, population-based cohort design; had a long follow-up duration, and an examined data from large number of patients with CHDs, even if patients were treated at different hospitals. To the best of our knowledge, no prior research has explored fertility in women with CHDs.

In conclusion, this pilot research revealed that CHDs compromises live births and female fertility, even for simple forms of CHDs. For all forms of CHDs, it is recommended that pregnant patients should be appeared to and advised individually by multidisciplinary team of pediatric cardiologists, obstetricians, and anesthetists with relevant expertise.

## Data Availability Statement

The original contributions presented in the study are included in the article, further inquiries can be directed to the corresponding author.

## Ethics Statement

The studies involving human participants were reviewed and approved by Institutional Review Board of Chang Gung Medical Foundation (IRB No. 1906140058). Written informed consent from the participants' legal guardian/next of kin was not required to participate in this study in accordance with the national legislation and the institutional requirements.

## Author Contributions

S-JC, Y-JL, M-HL, and C-FH contributed to conception and design of the study. S-JC organized the database and wrote the first draft of the manuscript. Y-HY performed the statistical analysis. All authors contributed to manuscript revision, read, and approved the submitted version.

## Conflict of Interest

The authors declare that the research was conducted in the absence of any commercial or financial relationships that could be construed as a potential conflict of interest.
